# LAT1 (SLC7A5) catalyzes copper(histidinate) transport switching from antiport to uniport mechanism

**DOI:** 10.1016/j.isci.2023.107738

**Published:** 2023-08-26

**Authors:** Raffaella Scanga, Mariafrancesca Scalise, Nadia Marino, Francesco Parisi, Donatella Barca, Michele Galluccio, Chiara Brunocilla, Lara Console, Cesare Indiveri

**Affiliations:** 1 Department DiBEST (Biologia, Ecologia, Scienze della Terra) Unit of Biochemistry and Molecular Biotechnology, University of Calabria, 87036 Arcavacata di Rende, Italy; 2 MAT-INLAB (Laboratorio di Materiali Molecolari Inorganici), Department of Chemistry and Chemical Technologies (CTC), University of Calabria—UNICAL, Via P. Bucci, 87036 Arcavacata di Rende, Italy; 3 Department DiBEST (Biologia, Ecologia e Scienze della Terra), 87036 Arcavacata di Rende, Italy; 4 CNR Institute of Biomembranes, Bioenergetics and Molecular Biotechnologies (IBIOM), 70126 Bari, Italy

**Keywords:** Chemistry, Inorganic chemistry, Molecular inorganic chemistry, Biochemistry

## Abstract

LAT1 (SLC7A5) is one of the most studied membrane transporters due to its relevance to physiology in supplying essential amino acids to brain and fetus, and to pathology being linked to nervous or embryo alterations; moreover, LAT1 over-expression is always associated with cancer development. Thus, LAT1 is exploited as a pro-drug vehicle and as a target for anti-cancer therapy. We here report the identification of a new substrate with pathophysiological implications, i.e., Cu-histidinate, and an unconventional uniport mechanism exploited for the Cu-histidinate transport. Crystals of the monomeric species Cu(His)_2_ were obtained in our experimental conditions and the actual transport of the complex was evaluated by a combined strategy of bioinformatics, site-directed mutagenesis, radiolabeled transport, and mass spectrometry analysis. The LAT1-mediated transport of Cu(His)_2_ may have profound implications for both the treatment of copper dysmetabolism diseases, such as the rare Menkes disease, and of cancer as an alternative to platinum-based therapies.

## Highlights

• Copper-histidinate is an additional substrate of the plasma membrane transporter LAT1
• The transport of Cu(His)_2_ requires binding of internal ATP at the residue K204
• The uptake of Cu(His)_2_ occurs with a uniport mode of transport
• Cu(His)_2_ transport may have implications in treatment of Menkes Disease and cancer

## Graphical abstract

**Figure:**
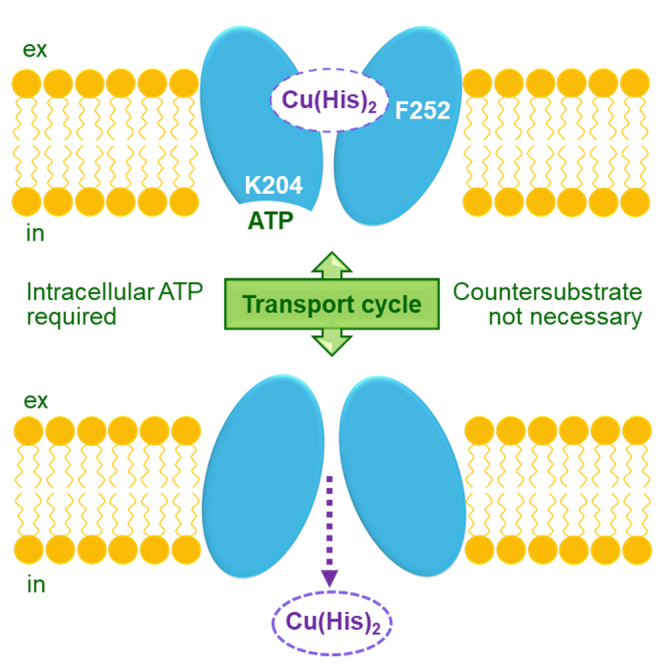

## Introduction

LAT1 (L-type amino acid transporter 1) is a well-characterized transporter for essential amino acids (EAAs), with a high preference toward histidine and leucine.^1^^,^^2^ Due to its localization in the plasma membrane of endothelial cells in the placenta and blood-brain barrier (BBB), LAT1 has an essential role in fetus growth and normal brain development.^3^^,^^4^^,^^5^^,^^6^ Indeed, the KO embryos for murine LAT1 are not vital^7^ and the intrauterine growth restriction (IUGR) syndrome is characterized by low levels of EAA in the placenta.^8^ Furthermore, point mutations affecting LAT1 function and transport mechanism are causative of some familiar cases of autism spectrum disorders (ASDs).^9^ In these patients, LAT1 defects are associated with an abnormal brain accumulation of histidine and very low levels of the other EAAs, well correlating with the substrate preference of the transporter.^9^ However, the current terrific interest toward LAT1 is related to its well-documented over-expression in virtually all human cancers.^6^^,^^10^ Indeed, LAT1 provides cancer cells with EAAs required for *de novo* protein synthesis, metabolic changes and signaling pathway(s).^11^^,^^12^ Over the years, the mentioned findings boosted the pharmaceutical research to target LAT1 for improving anticancer therapies.^13^^,^^14^^,^^15^^,^^16^^,^^17^ The efforts of several research groups worldwide allowed designing a tyrosine analogue, JPH203, which reached the clinical trial for solid tumors.^18^ At a molecular level, the peculiarity of LAT1 is its association with a glycoprotein belonging to the SLC3 family, namely CD98 (SLC3A2). The data collected over the years established that the non-glycosylated LAT1 is the sole transport competent unit of the heterodimer,^19^ whereas the glycosylated CD98 is responsible for routing LAT1 to the cell plasma membrane.^20^ In particular, the four N-glycosyl residues of CD98 have a major role in LAT1 trafficking.^21^

The transport features of LAT1 have been studied in intact cell systems and proteoliposomes revealing an antiport sodium-independent mode that, physiologically, contributes to harmonizing the amino acid cell pools.^1^^,^^2^^,^^22^ Interestingly, LAT1 transport function is positively regulated by the physical interaction with cholesterol.^23^^,^^24^ Moreover, upon binding of cholesterol, LAT1 can interact with ATP via an internal pocket of the protein close to the substrate binding site, triggering a further stimulation of the transport activity.^23^ The presence of LAT1 at the BBB inspired several studies in the design of prodrug, i.e., a pharmacological compound that, upon administration, releases a pharmacologically active drug. This approach is devoted to improving the absorption and distribution of an administered drug, particularly in those body districts, of which BBB is an eminent example, where the drug permeability is very poor.^25^ In this respect, LAT1 is considered a good trojan horse for treating neurological disorders using its substrate as a scaffold of drugs.^26^^,^^27^ In the list of neurological disorders, a rare disease attracted our attention, the Menkes Disease (MD), also known as kinky hair syndrome (OMIM: 309400). This is a rare genetic disorder that strongly impairs copper uptake, distribution and utilization in the body.^28^^,^^29^ Copper, like zinc and iron, is an essential trace metal acting as a cofactor of several enzymes.^30^ Copper enters enterocytes by a specific membrane transporter, namely CTR1, and is then pumped out from cells through P-type pumps ATPase7A and ATPase7B to reach the other tissues.^31^ In the blood, copper is bound to chaperones such as albumin and ceruloplasmin. The gene encoding for ATPase7A is located on chromosome X and diverse mutations, up to 370, are causative of the onset of MD with a variable degree of severity and symptoms. Classical MD and its milder allelic variant, occipital horn syndrome (OHS), are characterized by progressive cerebral and cerebellar neurodegeneration, intellectual disabilities, fair skin with kinky hair and connective tissue disturbances.^28^ Symptoms of the disease are mostly associated with the malfunctioning of copper-dependent enzymes, such as dopamine-β-hydroxylase, responsible for dopamine synthesis. So far, no treatment exists for MD, except for subcutaneous or intravenous administration of copper histidine.^28^^,^^32^ The patients affected by OHS are also treated with copper histidine even though the efficacy is not definitively assessed.^33^ The efficacy of treatments has been linked to two major factors: (1) proper timing, i.e., the treatment has to be provided prior to the symptoms manifestation, and (2) mutations of ATPase7A which are not disruptive of the copper-transport function. Therefore, current treatments are not generally effective, and the disease is often lethal for newborns. Given the ability of copper to coordinate several atoms, including nitrogen, oxygen and sulfur, the conjugation with amino acid has been exploited as a route for providing cells with copper. In particular, a clinical trial has been realized using copper-histidinate (NCT00001262) as the copper supplement.^34^ L-histidine is a potential tridentate ligand, whose coordination mode(s) are strongly affected by pH, metal-to-ligand ratio, and not least the specific nature of the coordinating metal ion. Indeed, the speciation of the copper(II)-L-histidine system in aqueous solutions has been the subject of a multitude of studies for decades.^35^^,^^36^^,^^37^ Pertinent to this work, literature data strongly suggest the occurrence of nearly one complex species around physiological pH in aqueous solution (where the free histidine amino acid exists predominantly in its neutral form, but any metal(II)-His complex would feature the monoanionic histidinate ion), i.e., a copper(II)-bis(L-histidinate) monomer, regardless of the synthetic stoichiometric His:Cu^2+^ ratio being closer to 1:1 or 2:1.^35^^,^^37^ In this frame, considering the specificity of LAT1 toward histidine^19^^,^^22^^,^^38^ together with its sensitivity to divalent heavy metals, such as mercury,^39^ and its localization at the BBB and at the placenta barriers,^5^ we sought to investigate the possible interaction of LAT1 with copper. We report surprising experimental findings by complementary experimental approaches, shedding new light on the plausible molecular mechanism of cell absorption of copper in complex with histidine. The described results represent a milestone for implementing and/or improving human therapies for diseases characterized by alterations in copper absorption, distribution and utilization.

## Results

### Effect of Cu^2+^ on LAT1 transport function

**Figure 1:**
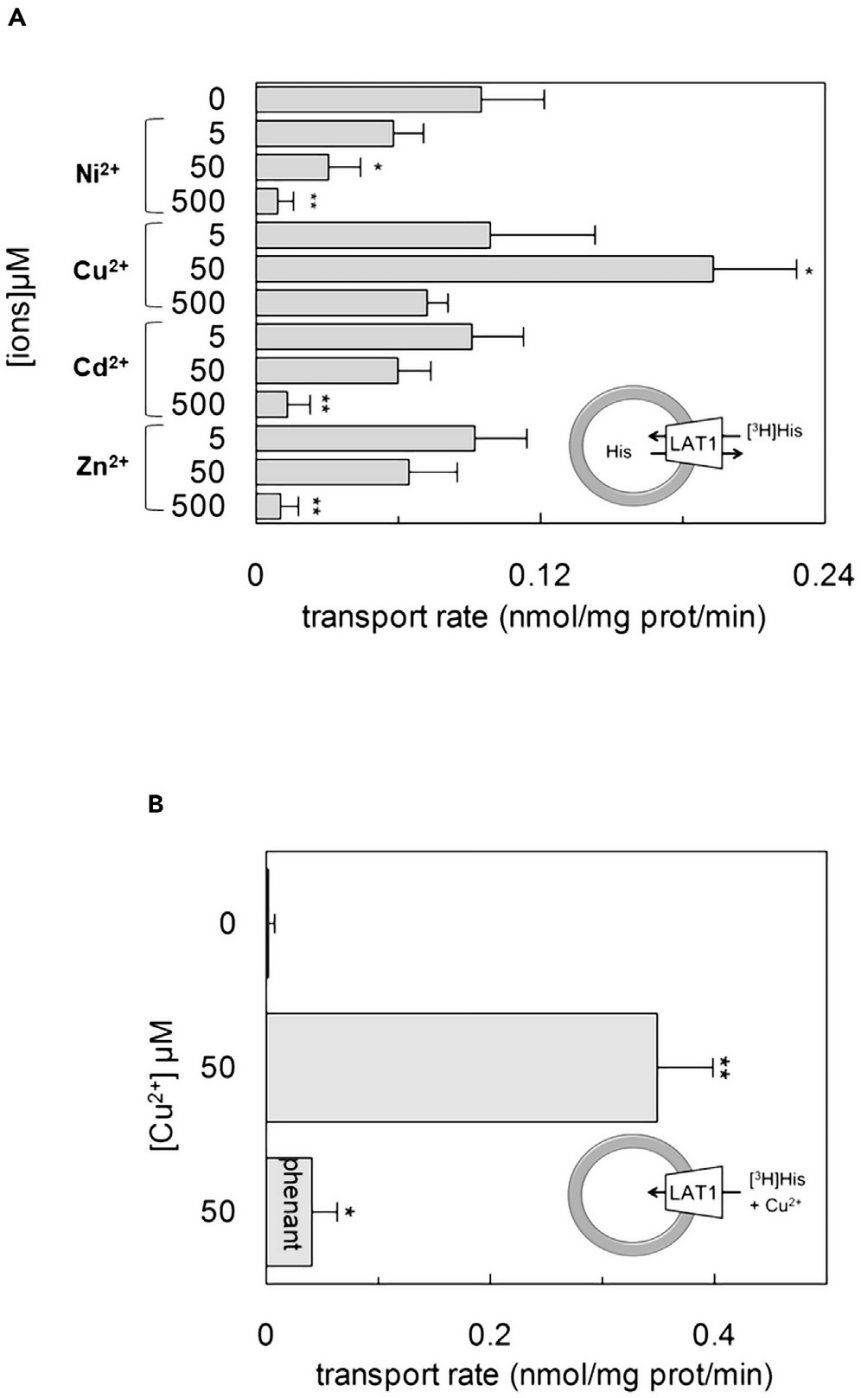
Effect of divalent cations on hLAT1 reconstituted in proteoliposomes The reconstitution was performed as described in STAR Methods. (A and B) In (A), the transport was measured by adding 5 μM [^3^H]-histidine to proteoliposomes containing 10 mM histidine in the presence of different divalent cations: Ni ^2+^, Cu ^2+^, Zn ^2+^, Cd ^2+^ at concentrations 5, 50 and 500 μM added as nichel(II) sulfate, copper(II) sulfate, zinc(II) chloride and cadmium(II) chloride. In (B), the uptake of 5 μM [^3^H]-histidine, to proteoliposomes without internal substrate, was measured in the absence or presence of 50 μM of copper(II)sulfate and 100 μM phenanthroline. The transport was stopped after 20 min according to the stop inhibitor method as described in STAR Methods. The transport rate was expressed as nmol/mg/min. Results are means ± S.D. from three independent experiments significantly different from control sample (none) as estimated by Student’s *t* test (∗p < 0.05).

The effect of some metal ions, chosen among endogenous or exogenous, was tested on the transport function of LAT1 reconstituted in proteoliposomes. This single transporter *in vitro* system allows dissecting of the specific effect of metals on LAT1. Not surprisingly, Ni^2+^, Cd^2+^, or Zn^2+^ exerted concentration dependent inhibition of the [^3^H]-histidine_ex_/histidine_in_ antiport, probably by interacting with one or more Cys residues of the protein, as in the case of Hg^2+^.^2^^,^^39^ Interestingly, Cu^2+^ showed a different behavior: it did not exert any effect at 5 μM; it triggers a strong stimulation of transport activity at 50 μM; a slight, if any, inhibition was observed only at 500 μM (Figure 1A). As stated in the introduction, LAT1 is an obligatory antiporter of amino acids, then, to further investigate the stimulatory effect exerted by Cu^2+^, the accumulation of [^3^H]-histidine was measured also in the absence of internal histidine, i.e., under a condition in which LAT1 is not active (Figure 1B).^19^^,^^40^ Intriguingly, the presence of Cu^2+^ was able to stimulate [^3^H]-histidine uptake even without internal substrate (Figure 1B). As expected, the uptake of [^3^H]-histidine, in the absence of Cu^2+^, was negligible instead. Accordingly, the copper(II)-induced uptake of [^3^H]-histidine was suppressed by the copper-chelating agent phenanthroline.

**Figure 2:**
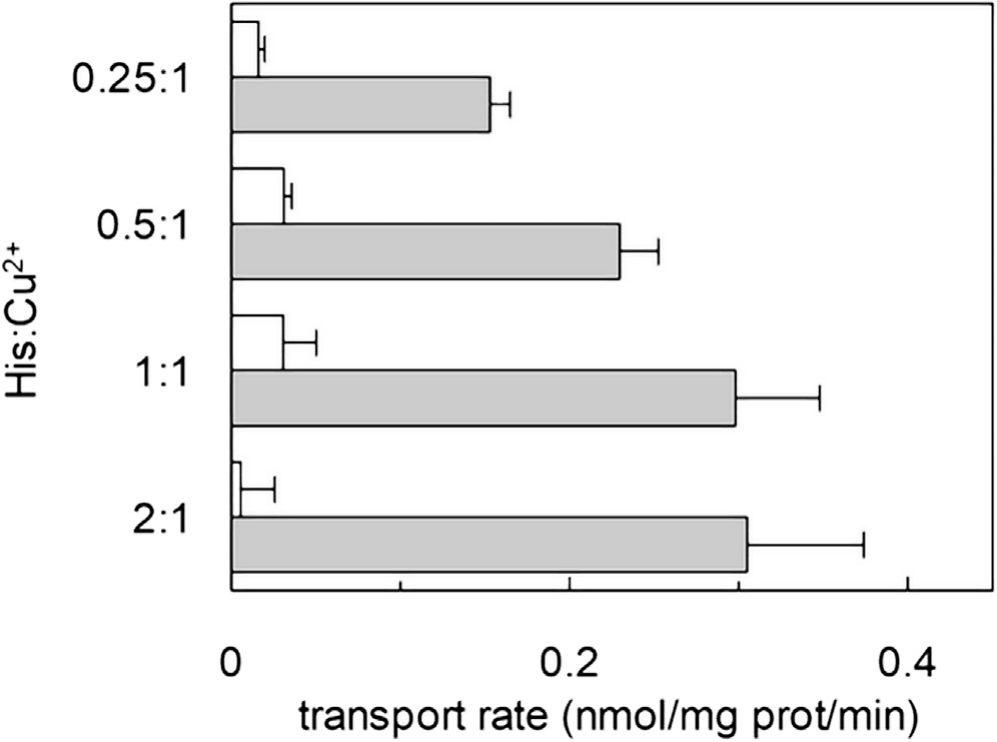
Dependence of the transport activity of hLAT1 in proteoliposome on Histidine/Copper(II) ratio The reconstitution was performed as described in STAR Methods. The transport was measured by adding 5, 10, 20 or 40 μM [^3^H]-histidine in the presence of 20 μM of copper(II)sulfate to proteoliposomes in the absence of internal substrate. Gray bars represent the samples prepared in the presence of intraliposomal 4 mM ATP; white bars represent the samples prepared in the absence of internal ATP. The transport was stopped after 20 min according to the stop inhibitor method as described in STAR Methods. The transport rate was expressed as nmol/mg/min. Results are means ± S.D. from three independent experiments. Multiple comparisons were performed for condition with and without internal ATP, using non parametric Kruskal-Wallis test and no difference between pair of groups have been estimated.

The [^3^H]-histidine uptake was then measured under different His:Cu^2+^ ratios, in the absence of internal histidine (Figure 2). The results showed that the accumulation of [^3^H]-histidine increases by increasing the His:Cu^2+^ ratio. In the same experiments, another feature of LAT1 transport was evaluated, i.e., the effect of intraliposomal ATP that stimulates the physiological transport activity.^23^ Interestingly, the copper(II)-coupled [^3^H]-histidine accumulation was strongly stimulated by internal ATP (Figure 2). This phenomenon was particularly pronounced for the 2:1 stoichiometry; in this case, virtually no transport was measured in the absence of intraliposomal ATP. Altogether, the data describe the capacity of LAT1 to switch from an antiport mechanism to a uniport one which is only observed in the presence of internal ATP and external Cu^2+^.

### Specificity of LAT1 in mediating Cu^2+^-coupled [^3^H]-histidine uptake

**Figure 3:**
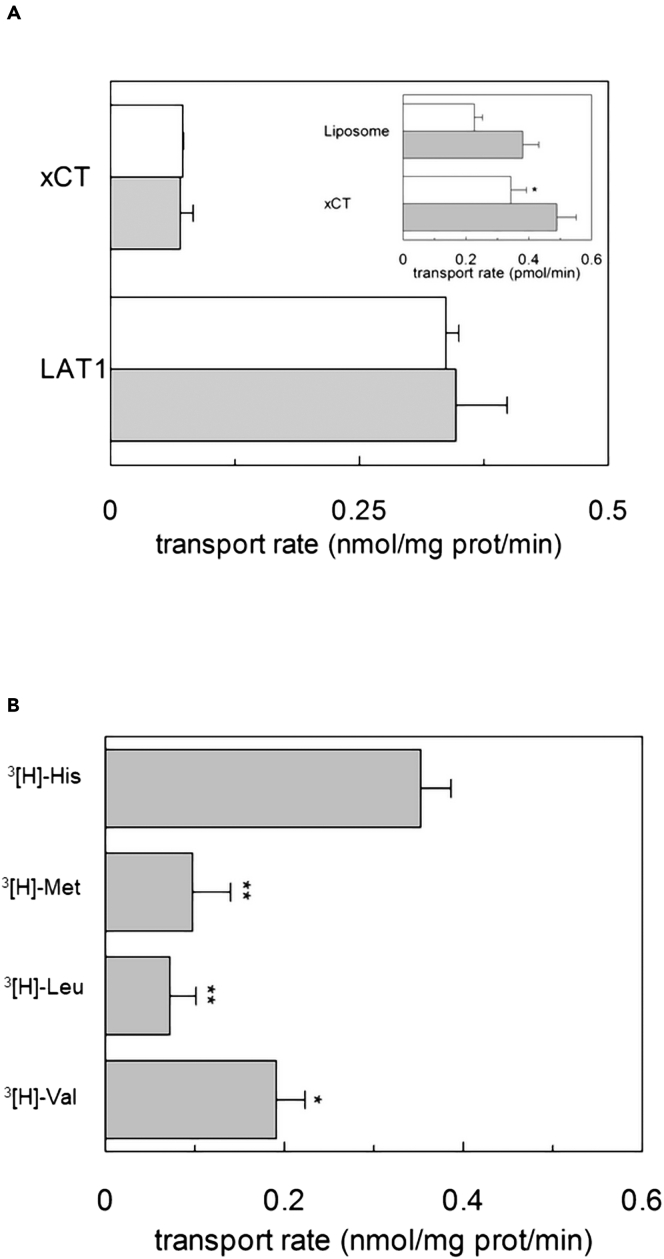
Evaluation of [^3^H]-histidine uptake in proteoliposomes reconstituted with hLAT1 or hxCT (A and B) In (A), the purified proteins hxCT or hLAT1, were reconstituted as described in STAR Methods. The transport was measured by adding 40 μM [^3^H]-histidine and 20 μM copper(II)sulfate (gray bars) or 20 μM [^3^H]-histidine and 20 μM copper(II)sulfate to proteoliposomes (white bars), prepared in the absence of internal histidine and in the presence of 4 mM ATP. The transport was stopped after 20 min according to the stop inhibitor method as described in STAR Methods. The transport rate was expressed as nmol/mg/min. Results are means ± S.D. from three independent experiments. In the inset of the figure, a significant difference from control sample (empty liposome), as estimated by Student’s *t* test (∗p < 0.05). In (B), the reconstitution was performed as described in STAR Methods. The transport was measured by adding 40 μM [^3^H]-histidine, 40 μM [^3^H]-leucine, 40 μM [^3^H]-valine or 40 μM [^3^H]-methionine, in the presence of 20 μM copper(II)sulfate to proteoliposomes prepared in the absence of internal substrate and in the presence of 4 mM ATP. The transport was stopped after 20 min according to the stop inhibitor method as described in STAR Methods. The transport rate was expressed as nmol/mg/min. Results are means ± S.D. from three independent experiments significantly different from control sample ([^3^H]-histidine transport) as estimated by Student’s *t* test (∗p < 0.05).

To evaluate the specificity of the above-described phenomenon, another membrane protein belonging to the same SLC7 family and characterized by an antiport mode of transport as LAT1, i.e., the human xCT (SLC7A11), was reconstituted under the same conditions of LAT1 and tested for sensitivity to Cu^2+^ (Figure 3A). Interestingly, the uptake of the copper-coupled [^3^H]-histidine, at His:Cu^2+^ 1:1 and 2:1 ratios, was detected only in proteoliposomes harboring LAT1; whereas the accumulation of [^3^H]-histidine in proteoliposomes harboring xCT overlapped the unspecific diffusion in liposomes with no reconstituted protein (Figure 3A-inset). Since the phenomenon was LAT1 specific, different known substrates of the transporter were tested.^41^^,^^42^ In particular, [^3^H]-valine, [^3^H]-leucine, and [^3^H]-methionine were tested in parallel to [^3^H]-histidine, at a 2:1 ratio with Cu^2+^ in the presence of intraliposomal ATP (Figure 3B). Interestingly, the activation was much more evident when using [^3^H]-histidine, indicating a preference for histidine over the other known LAT1 substrates.

**Figure 4:**
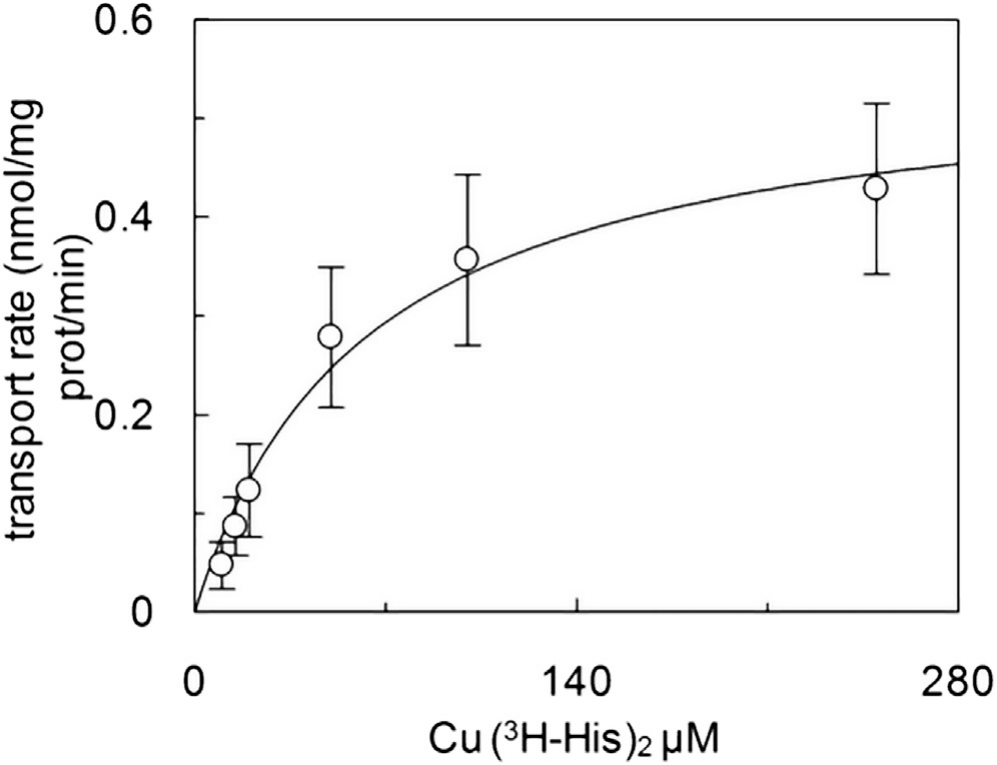
Kinetics of transport in hLAT1-reconstituted proteoliposomes The purified hLAT1 was reconstituted as described in STAR Methods. The transport rate was measured by adding [^3^H]-histidine in ratio 2:1, with copper(II)sulfate at the indicate concentrations, to proteoliposomes prepared in the absence of internal substrate and in the presence of 4 mM ATP. The transport was measured in 20 min according to the stop inhibitor method. The transport rate was expressed as nmol/mg/min. Data were plotted according to Michaelis-Menten equation. Results are means ± S.D. from three independent experiments.

Then, the kinetics of the 2:1 [^3^H]-histidine:copper(II) system was evaluated by measuring the uptake at increasing concentrations (Figure 4).

The data, fitted according to the Michaelis-Menten kinetics, gave a K_m_ of 61.0 ± 14.5 μM, which is close to the K_m_ measured for the canonical antiport reaction.^19^^,^^23^^,^^40^

### Crystallization of the 2:1 histidinate:Cu^2+^ complex and testing in the transport assay

**Figure 5:**
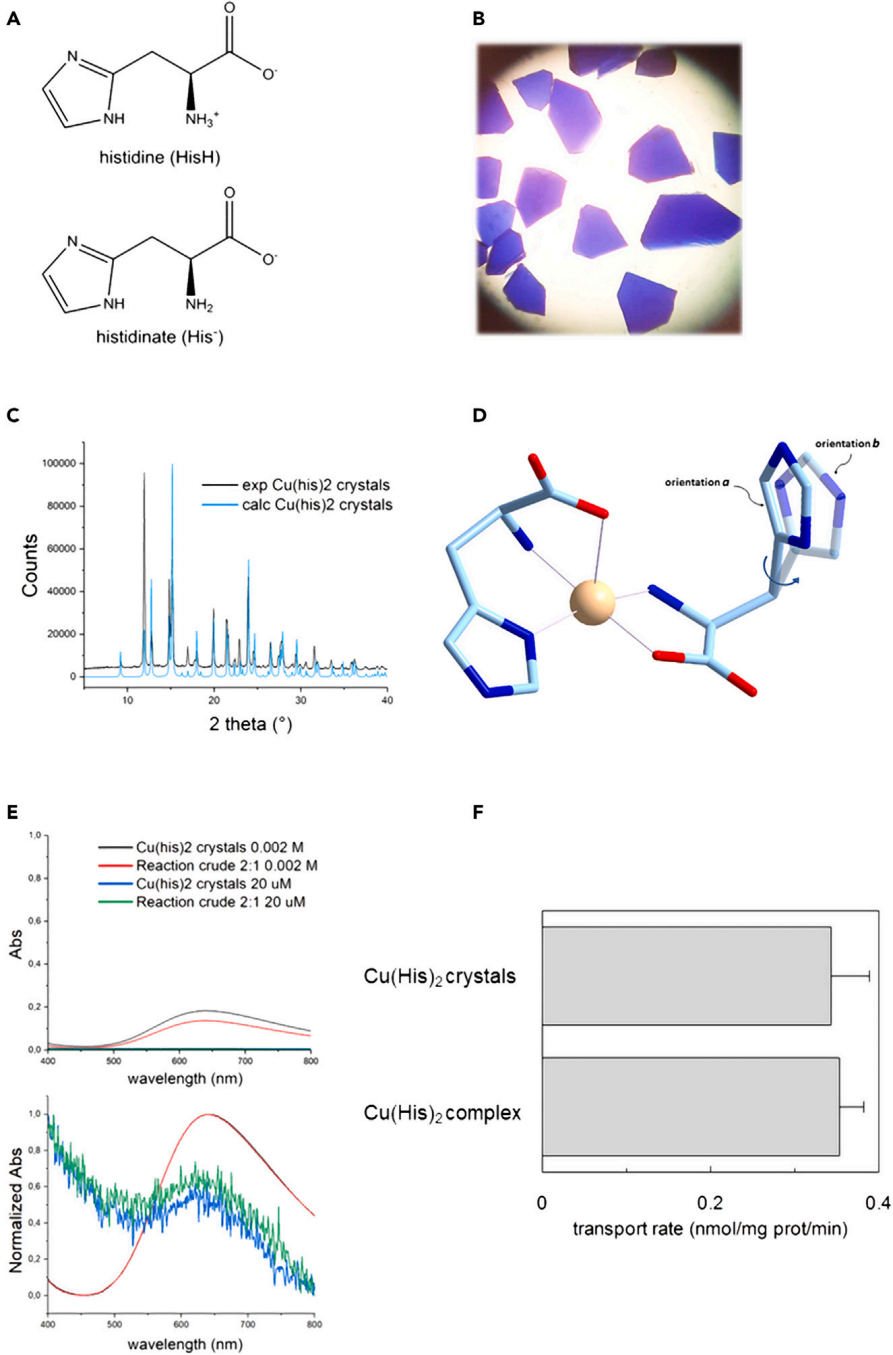
Crystals of Copper(II)histidinate and transport in LAT1-reconstituted proteoliposomes (A–F) In (A), schematic view of L-histidine at physiological pH (free AA, predominant protonation form, HisH) and of the histidinate anion at the same pH (metal-bound AA, HisH^−^). In (B), microscope image of crystals of [Cu(His)_2_]·1.5H_2_O obtained in this work. In (C), PXRD profiles of the grinded crystals of [Cu(His)_2_]·1.5H_2_O obtained in this work, compared to the theoretical PXRD pattern calculated from the literature available single-crystal data (CCDC Refcode EBAJUN). In (D), a view of the molecular structure of the Cu(His)_2_ complex in the crystal structure of [Cu(His)_2_]·1.5H_2_O^43^ [color codes: Cu, pale pink; C, light blue; O, red; N, blue; H, not shown]. Only the structural disorder involving the pendant imidazole group, but not that involving the coordinated N atom of the same ligand is shown, for clarity]. In (E), electronic absorption spectra (top: Abs; bottom: normalized Abs) of either dissolved [Cu(His)_2_]·1.5H_2_O crystals or the reaction crude (His + CuSO_4_, 2:1) in PBS (1×, without NaCl, pH 7.2) at 298 K, at concentrations of about 2 mM and 20 μM. The panels A-E are available at larger size in the supplemental information. In (F), the purified hLAT1 was reconstituted as described in STAR Methods. The transport rate was measured by adding 40 μM [^3^H]-histidine with 20 μM copper(II)sulfate or with 20 μM Cu(His)_2_ complex solubilized from crystals, to proteoliposomes prepared in the absence of internal substrate and in the presence of 4 mM ATP. The transport was measured in 20 min according to the stop inhibitor method. The transport rate was expressed as nmol/mg/min. Results are means ± S.D. from three independent experiments non-significantly different from control sample (Cu(His)_2_ complex) as estimated by Student’s *t* test (p < 0.05).

Based on the described results, bearing in mind the literature background, we hypothesized that a Cu(His)_2_ complex rapidly self-assemblies *in situ* under our experimental conditions, at least at the 2:1 His:Cu^2+^ stoichiometry. Therefore, we sought to investigate the possibility to pre-form and characterize the complex at the solid-state, then test it directly for transport by LAT1 reconstituted in proteoliposomes. A neutral, monomeric complex of copper(II) and L-histidinate with a His:Cu^2+^ 2:1 stoichiometry could be effectively prepared and crystallized at pH 7.3–7.4, following the pioneering procedure of Sarkar et al., which in 2004 succeed in a task that had eluded many researchers around the world for over 40 years^35^^,^^43^ (Figures 5A and 5B). The obtained sample was characterized at the solid-state by a combination of spectral and diffraction analyses. FT-IR features were congruent with the literature (ν_as_(CO_2_^−^) centered around 1600 cm^−1^, ν_s_(CO_2_^−^) = 1395 cm^−1^, ν(imidazole) = 1113 cm^−1^) (Figure S1).^43^ Single-crystal and powder X-ray diffraction (Figure 5C), confirmed both the previously reported crystal formulation in terms of solvent content, [Cu(His)_2_]·1.5H_2_O, and crystal structure, including the static disorder of the unbound imidazole group (showing two preferential orientations with 50:50 occupancy) (Figure 5D).^43^ The UV-vis absorption profiles of the dissolved crystals in either aqueous solution or PBS were also consistent with the literature in a wide range of concentrations, including millimolar and micromolar (Figure 5E). Interestingly, the absorption profiles of the 2:1 reaction crude in the same conditions (solvent, concentration) and the latter were essentially superimposable (Figure 5E).

In line with these observations, the crystallized Cu(His)_2_ complex was solubilized and used in the transport assay in LAT1-harbouring proteoliposomes, in comparison to the *in situ* formed 2:1 complex (Figure 5F). Interestingly, the uptake of the pre-formed Cu(His)_2_ crystal and that of the *in situ* formed His:Cu^2+^ 2:1 were virtually identical.

### Identification of Cu(His)_2_ binding site by docking analysis and validation by site-directed mutagenesis

**Figure 6:**
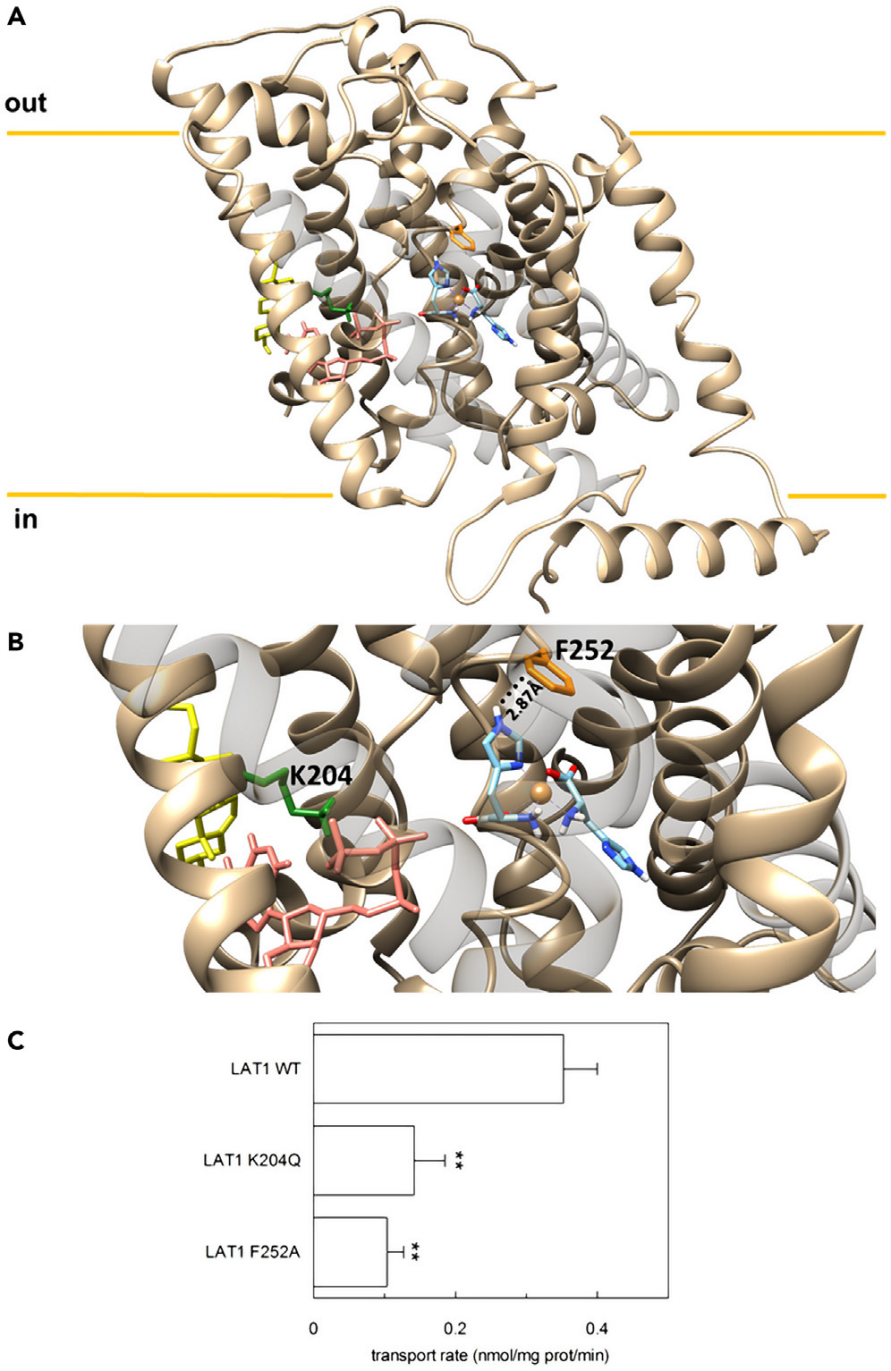
Molecular docking of Cu(His)_2_ and site-directed mutagenesis of hLAT1 (A–C) In (A), a ribbon representation of LAT1 (tan) in the inward open conformation with cholesterol (yellow) and ATP (salmon) bound, and in (B) a zoom of the binding site, are shown. The residues K204 (green), F252 (orange), and the complex Cu(His)_2_ (light blue) are highlighted. Molecular graphics and analyses were performed with UCSF Chimera, developed by the Resource for Biocomputing, Visualization, and Informatics at the University of California, San Francisco, with support from NIH P41-GM103311.^69^ In (C), the purified LAT1-WT, K204Q or F252A proteins were reconstituted as described in STAR Methods. The transport was started by adding 40 μM [^3^H]-histidine and 20 μM copper(II)sulfate to proteoliposomes in the absence of internal substrate and in the presence of 4 mM ATP. The transport was measured in 20 min according to the stop inhibitor method. The transport rate was expressed as nmol/mg/min. Results are means ± S.D. from three independent experiments significantly different from control sample (wild type) as estimated by Student’s *t* test (∗p < 0.05).

Moving from the obtained indications of a Cu(His)_2_ uptake, we performed *in silico* analysis to predict the site of interaction of the complex with LAT1. To this aim, we set out to perform our analysis by using the available X-ray structural data for the complex,^43^ moving from the work by Ginotra & Kulkarni who provided convincing pieces of evidences of the solution structure of the Cu(His)_2_ complex resembling that found by Deschamps et al. at the solid state.^36^ Docking analysis was performed by using the two datasets for the Cu(His)_2_ separately (i.e., considering one orientation of the disordered imidazole at a time, shown in Figure 5D), and LAT1 in the inward conformation (Figure 6A). No matter the starting condition, the pendant imidazole reaches the same equilibrium orientation at the end of the simulations. Moreover, the Cu(His)_2_ complex identifies a binding site close to the crucial residue for substrate gating, i.e., the F252 (Figure 6B),^2^ the shortest contact being 2.87 Å, and matches the site of histidine binding previously described.^23^^,^^44^ The Cu(His)_2_ binding site is also not too far from residue K204, involved in the interaction with ATP^23^ (Figure 6B). To deepen this aspect, the mutants F252A and K204Q were tested for their ability to mediate the transport of the Cu(His)_2_ complex. In good agreement with docking analyses, the F252A and K204Q mutants revealed a significant loss of Cu(His)_2_ transport activity with respect to the WT (Figure 6C).

### Influence of Lys204 on the ATP dependence of the Cu^2+^-coupled histidine uptake

**Figure 7:**
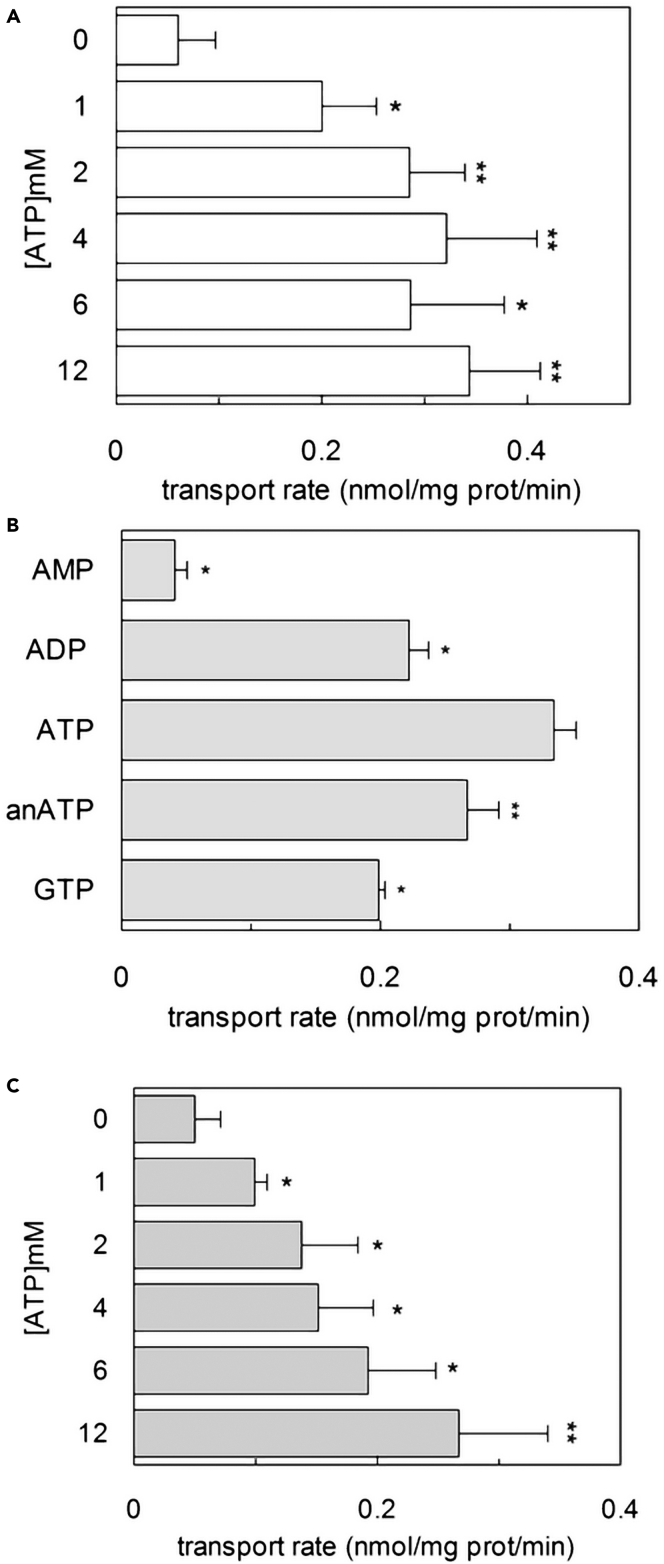
Effect of intraliposomal ATP on hLAT1 WT and hLAT1-K204Q mutant (A–C) The purified protein LAT1 WT (A-B) was reconstituted in proteoliposomes prepared as described in STAR Methods. The transport was started by adding 40 μM [^3^H]-histidine and 20 μM copper(II)sulfate to proteoliposomes reconstituted in the absence of internal substrate and in the presence of indicated concentrations of intraliposomal ATP (A) or 4 mM of intraliposomal AMP, ADP, ATP, anATP (the non-hydrolyzable ATP analogue, adenosine 5’-(β,γ-imido)triphosphate) or GTP (B). In (C), the purified protein LAT1 K204Q mutant was reconstituted in proteoliposomes prepared as described in STAR Methods. The transport was started by adding 40 μM [^3^H]-histidine and 20 μM copper(II)sulfate to proteoliposomes reconstituted in the absence of internal substrate and in the presence of indicated concentrations of intraliposomal ATP. In (A), (B) and (C) the transport was measured in 20 min according to the stop inhibitor method. The transport rate was expressed as nmol/mg/min. In (A) and (B), the transport was stopped by adding 5 mM of BCH; in (C), the transport was stopped by adding 5 mM of Pyridoxal 5 phosphate (PLP). Results are means ± S.D. from three independent experiments. In (A) and (C) significantly different from control sample (without internal ATP) as estimated by Student’s *t* test (∗∗p < 0.01; ∗p < 0.05). In (B), significantly different from sample with internal ATP 4 mM as estimated by Student’s *t* test (∗∗p < 0.01; ∗p < 0.05).

Considering the requirement for internal ATP, we evaluated the dependence of Cu(His)_2_ uptake on the internal ATP concentration (Figure 7A). The collected data showed that Cu(His)_2_ transport increases up to a plateau starting from 2 mM ATP. The ATP was specific since other nucleotides, including the non-hydrolyzable ATP analogue AnTP, were less effective (Figure 7B). Reported data are in good agreement with the effect of ATP on the canonical antiport [^3^H]-histidine_ex_:histidine_in_.^23^ Considering that Lys204 is the crucial residue for ATP binding,^23^ we employed the K204Q mutant to further assess the role of ATP in the Cu(His)_2_ transport (Figure 7C). The collected data showed that the transport activity at 2 mM ATP was much lower than that of WT, and the activity increased with increasing the ATP concentration up to 12 mM, without reaching a plateau as already observed for this mutant in the case of the canonical antiport [^3^H]-histidine_ex_/histidine_in_.^23^

### Cu^2+^-coupled histidine uptake in proteoliposomes mediated by native LAT1

**Figure 8:**
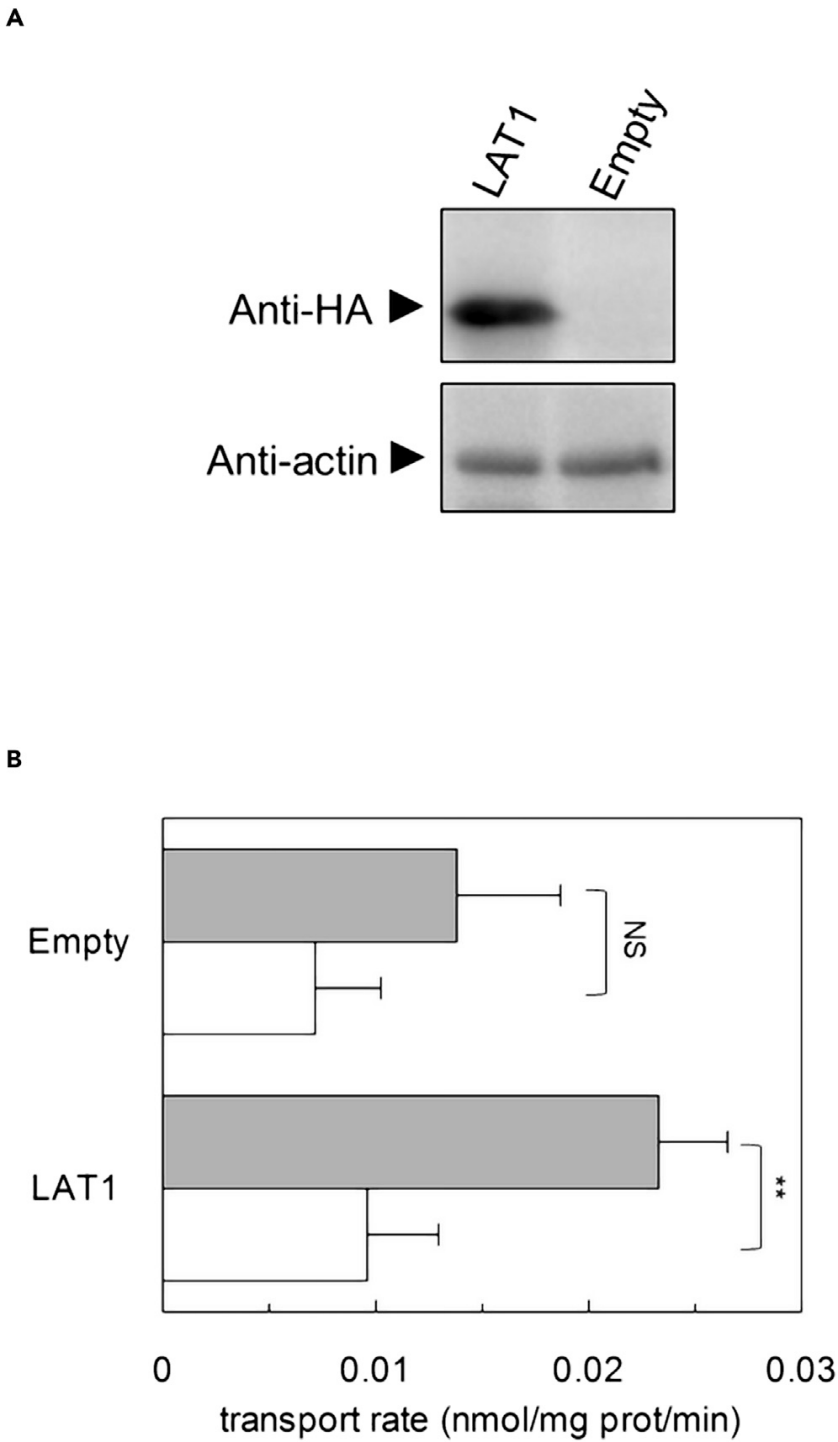
Evaluation of [^3^H]-histidine uptake in proteoliposomes reconstituted with hLAT1 overexpressed in HEK293-F (A and B) In (A), western blot analysis of HEK293-F transfected with empty plasmid or with hLAT1-HA construct as described in STAR Methods, using the antibody against the HA-tag. Anti-actin antibody was used as a loading control. In (B), HEK293-F cell lysates derived from empty vector or LAT1-HA construct were reconstituted in liposomes as described in STAR Methods. The transport was started by adding 40 μM [^3^H]-histidine and 20 μM copper(II)sulfate to proteoliposomes reconstituted in the absence of internal substrate and in the presence of 4 mM ATP. The transport was performed at room temperature (gray bars) or at 0°C (white bars). The transport was stopped after 20 min according to the stop inhibitor method as described in STAR Methods. The transport rate was expressed as nmol/mg/min. Results are means ± S.D. from three independent experiments, significantly different from control sample (room temperature) as estimated by Student’s *t* test (∗∗p < 0.01).

To ascertain if the same phenomenon was measurable also using the native LAT1, the transporter was over-expressed in HEK293 FreeStyle with an HA-tag (Figure 8A). Then, total lysates deriving from both empty vector and LAT1-HA-tag expressing cells were reconstituted in proteoliposomes for measuring the transport of the Cu(His)_2_ (Figure 8B). The uptake was more pronounced in proteoliposomes harboring LAT1 over-expressing lysates (Figure 8B). As a control, we performed the transport assay at 0°C to slow down transport. At this temperature, indeed, the transport mediated by the over-expressed LAT1 was significantly lower than the transport at room temperature, whereas in the case of lysates derived from empty vector transfected cells, the difference between room temperature and 0°C was not significant. Moreover, the slower transport at 0°C was very similar when comparing lysates derived from empty vector or over-expressing LAT1 cells (Figure 8B).

### Cu^2+^-coupled histidine uptake in intact cells

**Figure 9:**
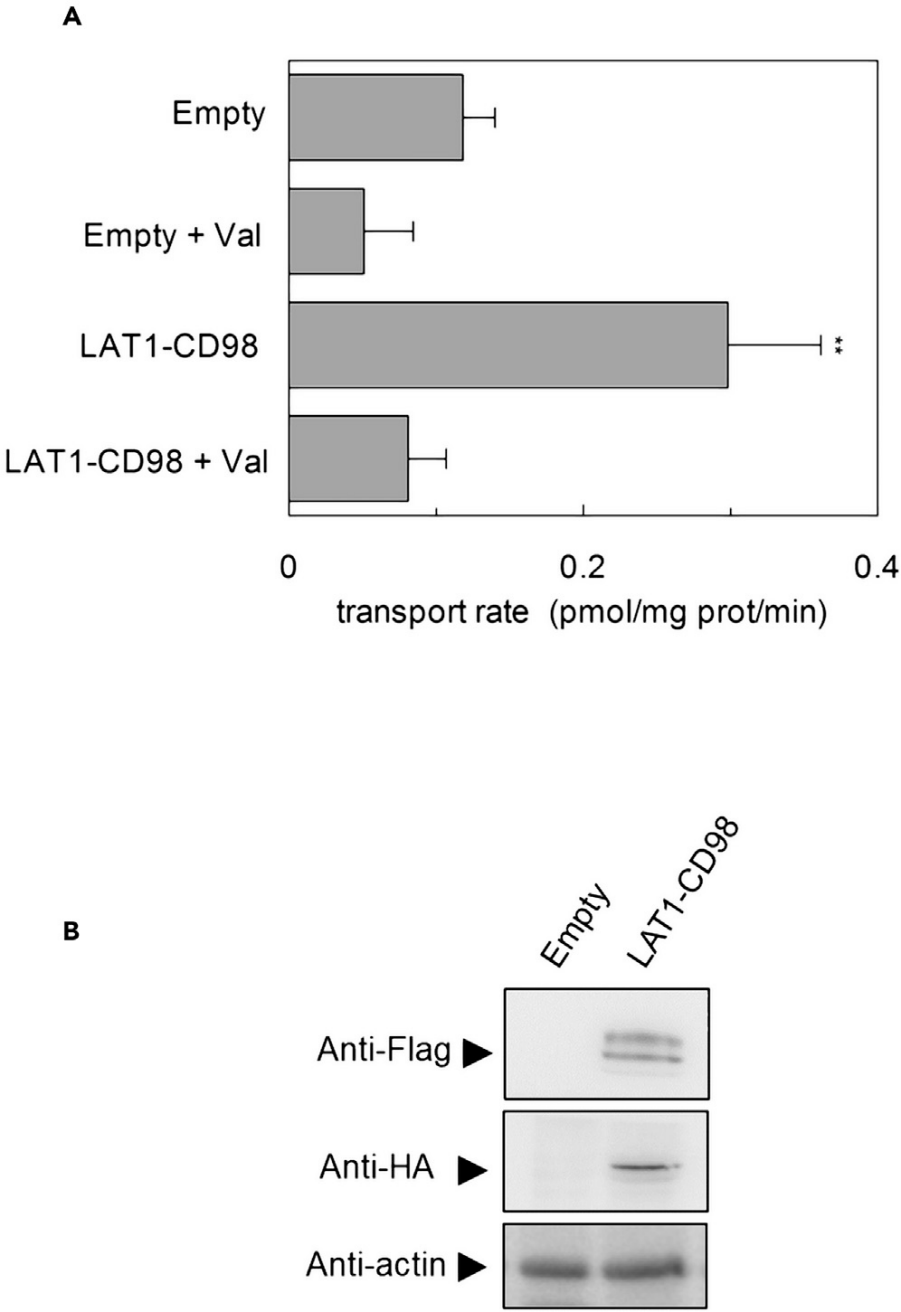
Evaluation of [^3^H]-histidine uptake in intact cells (A and B) In (A), HEK293 cells were transiently transfected with empty vector or with LAT1-HA and CD98-FLAG as described in STAR Methods. The transport was started adding 40 μM [^3^H]-histidine and 20 μM copper(II)sulfate in HBSS buffer at 37°C. The transport was stopped after 1 min with cold HBSS buffer on ice. The net LAT1-mediated transport was calculated by subtracting [^3^H]-histidine uptake in blank samples prepared with 10 mM of BCH. The specificity of LAT1-mediated transport was evaluated by adding 10 mM extracellular valine. Cells were lysed with 1% TX-100 and radioactivity was counted as described in STAR Methods. The transport rate was expressed as pmol/mg/min. Results are means ± S.D. from three independent experiments, significantly different from control sample (empty vector) as estimated by Student’s *t* test (p < 0.01). In (B), Western blot analysis of cell extracts of HEK293 transfected with empty vector or with LAT1-HA and CD98-FLAG constructs as described in STAR Methods, using the antibodies against the FLAG tag (upper panel), the HA-tag (intermediate panel) and actin used as loading control (lower panel).

HEK293 intact cells were transiently transfected with LAT1-HA, and Cu(His)_2_ accumulation was evaluated (Figure 9A). Considering the requirement for CD98 to allow LAT1 to properly reach the plasma membrane,^21^ we transfected CD98-FLAG together with LAT1-HA before transport measurement (Figure 9B). As shown in Figure 9A, the accumulation of Cu(His)_2_ was significantly higher in the cells transfected with LAT1-CD98 with respect to cells transfected with empty vector. The specificity of LAT1-mediated transport was also demonstrated by the inhibition observed upon adding Val, an acknowledged LAT1 substrate, to cells during the transport assay (Figure 9A).

### Measurement of Cu^2+^ transport in proteoliposomes

**Figure 10:**
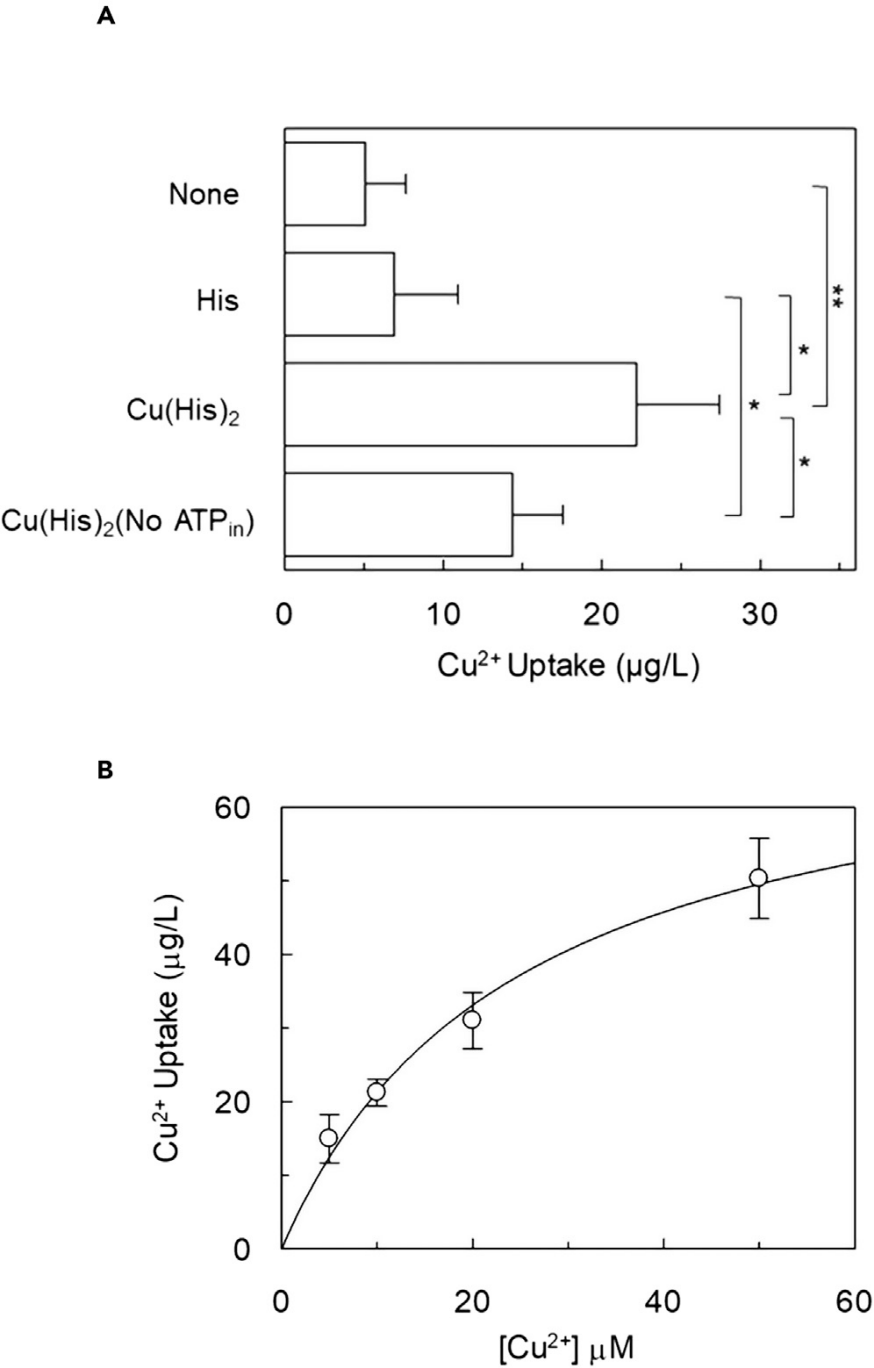
Evaluation of Copper uptake in proteoliposomes reconstituted with LAT1 The purified LAT1 was reconstituted in proteoliposomes prepared as described in STAR Methods. (A and B) In (A), the transport was started by adding 40 μM histidine and 20 μM copper(II)chloride to proteoliposomes reconstituted in the absence of internal substrate and in the presence of intraliposomal 4 mM ATP. The transport was measured in 60 min according to the stop inhibitor method. Five samples for each condition were pulled together, lysed with 0.25% C_12_E_8_ and subjected to ICP-MS analyses for copper quantification. The copper uptake was indicated as μg/L. Results are means ± S.D. from three independent experiments, significantly different as indicated in the figure, estimated by Student’s *t* test (∗∗p < 0.01, ∗p < 0.05). In (B), kinetics of copper uptake. The transport was started by adding indicated concentrations of histidine and copper(II)chloride, in a 2:1 ratio, to proteoliposomes reconstituted in the absence of internal substrate and in the presence of intraliposomal 4 mM ATP. The transport was measured in 30 min according to the stop inhibitor method. Five samples for each condition were pulled together, lysed with 0.25% C_12_E_8_ and subjected to ICP-MS analyses for copper quantification. The copper uptake was indicated as μg/L. Results are means ± S.D. from three independent experiments.

To check for evidence of copper being internalized together with histidine, an ICP-MS methodology was employed to directly measure the Cu^2+^ flux into LAT1-reconstituted proteoliposomes. When the complex Cu(His)_2_ is added to proteoliposomes, a much higher accumulation of Cu^2+^ is measured with respect to the conditions of controls (Figure 10A). Interestingly and in line with the above-described results, in the absence of intraliposomal ATP a significant lower accumulation of Cu^2+^ could be detected through ICP-MS analysis. To further address this point, the kinetics of copper accumulation was evaluated by ICP-MS; then, different concentrations of histidine:copper(II) complex were tested in a ratio 2:1 (Figure 10B). The data, fitted according to the non-linear Michaelis-Menten kinetics, gave a K_m_ of 25.4 ± 9.2 μM, which is about half the Km of Histidine (Figure 4) correlating well with the transport of the complex as well as with the 2:1 stoichiometry.

## Discussion

The role of LAT1 as a prodrug transporter was proposed for a long time^45^ and is still taken into great consideration.^40^^,^^46^ This serendipity function of LAT1 is related to the peculiar conformation of the substrate binding site, that on the one hand is highly selective for molecules harboring an amino acid functional group; on the other hand, it tolerates relatively large and hydrophobic side groups.^6^^,^^47^ Therefore, the transporter offers the possibility to couple LAT1 amino acid canonical substrates to compounds with pharmacological activity to facilitate their delivery to cells. This feature is even more attractive owing to the localization of LAT1 at the BBB with possible application to neurological disorders. In this frame, our results allowed us to propose LAT1 as the route for absorbing copper in those pathological conditions characterized by altered copper distribution, such as the rare genetic MD. Very interestingly and differently from the common inhibition effect exerted by heavy metals on several membrane transporters,^48^^,^^49^^,^^50^^,^^51^^,^^52^ copper showed a unique behavior in activating the LAT1 mediated histidine accumulation in proteoliposomes reconstituted with the recombinant or the native protein and also in intact cells. The results here provided, demonstrate that this unexpected behavior specifically occurs via LAT1.

As stated in the introduction, available literature data suggest that at physiological pH only a Cu(His)_2_ neutral monomeric species forms; still, the exact shape of this one and only species, both in solution and in the solid state, has been debated for decades and some confusion persists nowadays.^36^^,^^53^ Our investigation and results here provided, confirm that from the stoichiometric 2:1 ratio, the Cu(His)_2_ complex can be either precipitated or crystallized almost quantitatively. According to the collected data, such a 2:1 complex can be transported by LAT1. In line with the described copper(II)-histidine coordination, the transport of Cu^2+^ by the major copper transporter CRT1 occurs via an interaction with a His-rich region.^31^ By using the available crystallographic data,^43^ we observed that the Cu(His)_2_ complex docked into the substrate binding site of LAT1 nearby the previously identified crucial residues^2^^,^^23^ F252 and K204. In particular, in our simulation, the complex is capable of establishing close contact with the residue F252 via the copper-bound imidazole ring, while plausibly interacting with residue K204 through water and ATP-mediated hydrogen bonds. The interaction of LAT1 with larger substrates is not a novelty; indeed, several substrate analogues and/or prodrugs have been found to interact and to be transported by LAT1.^19^^,^^47^^,^^54^ An important novelty of our data is that LAT1, in the presence of the Cu(His)_2_ complex, switches the transport mechanism from an obligatory antiport to a fast uniport. To our knowledge, this is the first evidence of a uniport transport mediated by LAT1, a protein universally considered an obligatory antiporter of amino acids.^4^ The antiport mechanism has been indirectly proposed for the transport of some drugs by measuring the efflux of radiolabeled leucine induced by the addition of extracellular gabapentin, acivin, and fenclonine.^47^ More recently, by employing LC-MS analysis also ketoprofen was shown to be delivered via prodrug at the BBB level, possibly involving the exchange for intracellular LAT1 substrates.^27^ However, it cannot be excluded that LAT1-mediated prodrug transport, previously detected in intact cells, could occur by a uniport mechanism, as well.^15^^,^^55^ Indeed, when measuring transport in intact cell systems, it is difficult, if not impossible, to discriminate between antiport and uniport due to the forbidden access to the cell interior. Noteworthy, the proteoliposome tool helps the overcoming of this disadvantage because the internal compartment can be well controlled.^56^ We are aware that the proteoliposome system does not reproduce the cell environment, but this simplified and single-protein experimental model allowed us to discover the Cu(His)_2_ LAT1-mediated transport and to characterize it at a molecular level. Notwithstanding, we have verified the occurrence of the uptake of the Cu(His)_2_ complex in intact cells overexpressing LAT1, even if in this system, it was not possible to accurately evaluate the mechanistic properties. Indeed, these experiments have been conducted as a “proof-of-concept analysis” to envision an application of the Cu(His)_2_ complex to be used for delivering copper to cells/tissues of individuals with altered copper homeostasis, such as MD patients. We cannot completely exclude that CTR1 could have a role in Cu-His transport; however, copper should be transported alone by CTR1, according to the 3D structure demonstrating that the ion interacts directly with His and Met residues of the binding site.^57^ In this work, a double methodological approach has been pointed out to measure in parallel the fluxes of histidine and copper; to follow the entrance of histidine our classical radiometric assay has been used, whereas for copper flux an ICP-MS analysis has been employed. Another aspect of the observed Cu(His)_2_ transport in proteoliposomes, is the specific requirement for intraliposomal ATP, once more in good agreement with a LAT1-mediated transport.^23^ However, differently from the physiological antiport of amino acids which is stimulated by internal ATP, the Cu(His)_2_ transport has an essential requirement for ATP (Figure 2). Even though the precise molecular mechanism needs to be further defined, we can speculate that ATP may stabilize one or more of the conformations of LAT1 during the transport cycle. The concentration of ATP giving the best transport activity well correlates with the average intracellular concentration of the nucleotide. This further confirms that the Cu(His)_2_ complex could be employed to deliver copper to cells. Besides MD and OHS, the supply of copper to cells may reveal useful also for other neurological disorders, including Parkinson’s disease and a subset of Alzheimer’s disease characterized by a low level of copper in serum and in the brain.^58^ Considering the difficulty for rare disorders to be deepened in terms of novel target(s) and/or novel drug(s), we are confident that this study, even though at the preclinical level, may shed novel light on the rare disorders linked to copper dysmetabolism. Finally, our data on the Cu(His)_2_ transport by LAT1 may have another potential outcome, considering its well-documented over-expression in human cancers.^10^^,^^13^ Indeed, metal complexes, such as platinum-based drugs, are considered the most effective anticancer drugs commercially available.^59^ However, several side effects are linked to these drugs, then safer alternatives are welcome; in this respect, some copper complexes have been designed and tested in the last ten years,^60^ with different degrees of efficiency as anti-cancer agents. In this frame, the transport of Cu(His)_2_ by LAT1 could be another way for supplying copper to cells, employing the chemical scaffold of a physiological molecule.^58^

### Limitations of the study

This study may have drawbacks linked to translation to clinical applications that are not dealt with at this stage.

## STAR★Methods

### Key resources table

**Table:** 

REAGENT or RESOURCE	SOURCE	IDENTIFIER
**Antibodies**		

Anti-HA, Mouse monoclonal	Merck	Cat# H3663; RRID: AB_262051
Anti-FLAG M2, Monoclonal	Merck	Cat# F1804; RRID: AB_262044
Anti-Actin	Merck	Cat# A2066; RRID: AB_476693
HRP Goat anti-Mouse IgG	LI-COR	Cat# 926-80010; RRID: AB_2721263
HRP Goat anti-Rabbit IgG	Thermo Fisher	Cat# 656120; RRID: AB_2533967

**Bacterial and virus strains**		

Rosetta (DE3)pLysS competent cell	Merck	Cat# 70956-M

**Chemicals, peptides, and recombinant proteins**		

Adherent cell transport buffer (HBSS: Hanks' Balanced Salt Solution)	Homemade	Sutter et al.^61^
PBS without NaCl pH 7.0	This paper	N/A
2-Amino-2-norbornanecarboxylic acid (BCH)	Merck	Cat# A7902; CAS:20448-79-7
Pyridoxal 5′-phosphate hydrate ≥98% (PLP)	Merck	Cat# P9255; CAS: 853645-22-4
L-Histidine	Merck	Cat# H6034; CAS:71-00-1
L-Leucine	Merck	Cat# L8912; CAS: 61-90-5
L-Valine	Merck	Cat# V0513; CAS:72-18-4
L-Methionine	Merck	Cat# M5308
L-Glutamic acid monosodium salt hydrate	Merck	Cat# G1626; CAS:142-47-2
L-Glutamine	Merck	Cat# G3126; CAS:56-85-9
[^3^H]-L-Histidine	Moravek	Cat#MT905
[^3^H]-L-Leucine	PerkinElmer	Cat# NET1166001MC
[^3^H]-L-Valine	Moravek	Cat# MT1654
[^3^H]-L-Methionine	PerkinElmer	Cat# NET061X250UC
Copper (II) sulfate	Merck	Cat#C1297; CAS:7758-98-7
Copper (II) chloride	Merck	Cat# 751944; CAS:7447-39-4
Copper(II)nitrate hemi(pentahydrate)	Merk	Cat# 223395; CAS:19004-19-4
Trace Elements in Water	Merk	Cat# NIST1643F;
Cholesterol	Merck	Cat# C3045 CAS:57-88-5
n-Dodecyl β-D-maltoside ≥98% (GC)	Merck	Cat# D4641; CAS:692227-93-6
1,4-Dithioerythritol	Merck	Cat# D8255; CAS: 6892-68-8
Amberlite XAD-4	Merck	Cat# 06444; CAS: 37380-42-0
Octaethylene glycol monododecyl etherc12e8	TCI	Cat# O0139; CAS:3055-98-9
L-α-Phosphatidylcholine from egg yolk, ∼60% (TLC)	Merck	Cat#61755; CAS: 8002-43-5
FreeStyle™™ MAX Reagent	Thermo Fischer	Cat# 16447-100
PolyJet *In Vitro* DNA Transfection Reagent	SignaGenLaboratories	Cat# SL100688
Opti-MEM™ I	Thermo Fischer	Cat# 31985062
FreeStyle™ Expression Medium	Thermo Fischer	Cat# 12338018
Folin-Ciocalteus Phenolreagens	Merck	Cat# F9252
Lowry	Homemade	Lowry et al.^62^
Protease inhibitor cocktail	Merck	Cat# P8340; CAS:66701-25-5
hLAT1-wt	Homemade	Cosco et al.^23^^,^ Galluccio et al.^63^
hLAT1-K204Q	Homemade	Cosco et al.^23^
hLAT1-F252A	Homemade	Napolitano et al.^2^^,^ Scanga et al.^40^
hxCT	Homemade	Galluccio et al.^64^
Nickel(II) sulfate hexahydrate	Merck	Cat# 227676; CAS: 10101-97-0
Cadmium chloride	Merck	Cat# 202908; CAS: 10108-64-2
Zinc Cloride	Merck	Cat#746355; CAS:7646-85-7
1,10-Phenanthroline monohydrate	Merck	Cat# P9375; CAS: 5144-89-8
Adenosine 5'-Triphosphoric Acid Disodium Salt	Merck	Cat# A3377; CAS:34369-07-8
Adenosine 5′-diphosphate ≥95% (HPLC)	Merck	Cat# 01905; CAS:58-64-0
Adenosine 5′-monophosphate disodium salt	Merck	Cat# 01930; CAS:4578-31-8
Adenosine 5′-(β,γ-imido)triphosphate lithium salt hydrate	Merck	Cat# A2647; CAS:25612-73-1
Guanosine 5′-triphosphate sodium salt hydrate	Merck	Cat# G8877; CAS:36051-31-7
Pico-Fluor Plus	PerkinElmer	Cat# 6013691
SuperSignal™ West Femto Maximum Sensitivity Substrate	Thermo Fisher	Cat# 34096
PBS	Thermo Fisher	Cat# 10010023

**Critical commercial assays**		

TGX Stain-Free™ FastCast™ Acrylamide Kit, 12%	Bio-Rad	Cat#1610185

**Experimental models: Cell lines**		

FreeStyle™ HEK293-F cells	Thermo Fischer	Cat# R79007
HEK-293	Lab Infection Cancer Biology,WHO/OMS Lyon,France. Dr.Tommasino	Accardi et al.^65^

**Experimental models: Organisms/strains**		

Rosetta (DE3)pLysS competent cell	Merck	Cat# 70956-M

**Recombinant DNA**		

pCDNA3-LAT1-HA	Homemade	Berthold et al.^66^
pSF-CMV-NEO-NH_2_-3XFLAG-CD98	Homemade	Console et al..^21^^,^ Berthold et al.^66^

**Software and algorithms**		

AutodockVina v1.1.2	Eberhardt et al.^67^^,^ Trott O et al.^68^	https://vina.scripps.edu/downloads/
USCF Chimera v1.14	Pettersen et al.^69^	https://www.cgl.ucsf.edu/chimera/download.html
Grafit v5.0.13 Erithacus Software,West Sussex,UK		http://www.erithacus.com/grafit/grafit_5_service_pack.htm
Image Lab v5.2.1 (Bio-Rad)		https://www.bio-rad.com/it-it/product/image-lab-software?ID=KRE6P5E8Z

### Resource availability

#### Lead contact

Further information and requests for resources and reagents should be directed to and will be fulfilled by the lead contact, Cesare Indiveri (cesare.indiveri@unical.it).

#### Materials availability

This study did not generate new unique reagents.

### Method details

#### Over-expression of hLAT1-HA in HEK293FS cells and in HEK293 cells

FreeStyle™ HEK293-F cells were cultured in suspension into FreeStyle™ 293 Expression Medium. Approximately 24 hrs before transfection, HEK293-F cells at 0.6 × 10^6^– 0.7 × 10^6^ cells/mL density have been cultured on 125 mL polycarbonate shake flasks on an orbital shaker platform rotating at 135 rpm at 37°C, 8% CO_2_ atmosphere. On the day of transfection, the cells had 90% viability and to ensure high transfection results, they were diluted to a density of 1 × 10^6^ cells/mL, as described by the manufacturer (Invitrogen). HEK293-F cells were transfected with the hLAT1-HA construct or with the empty plasmid, as control. In brief, 37.5 μg of plasmid and 37.5 μL of 293fectin reagent were mixed with 0.6 mL of Opti-MEM in separate tubes, combined, and incubated at room temperature for 10 minutes, as described by the manufacturer (Invitrogen). The DNA-293fectin mixture was pipetted into a 125 mL polycarbonate shaker flask containing 1 × 10^6^ cells/mL Freestyle 293-F cells. Cultures continued for 3 days at 37°C and 8% CO_2_ and were harvested by centrifuging for 5 minutes, 100 g at room temperature. HEK293 cells were maintained in Dulbecco's Modified Eagle Medium (DMEM) supplemented with 10% Fetal Bovine Serum (FBS), 1 mM glutamine and 1 mM sodium pyruvate. Cells were grown on 75 cm^2^ plates at 37°C in a humidified incubator and a 5% CO_2_ atmosphere. For transient transfection and transport, HEK293 cells were seeded onto 12 well plates and cultured using standard culturing conditions until they reached 80% confluence. HEK293 cells were transfected with Polyjet transfection reagent according to the manufacturer's procedures; in brief 0.25 μg of pCDNA3-LAT1-HA and 0.5 μg of pSF-CMV-NEO-NH_2_-3XFLAG-CD98, or 0.75 μg empty vectors were diluted in 38 μL of DMEM serum and antibiotics-free combined with 2.25 μL polyjet in 38 μL of DMEM serum and antibiotics-free. After 15 min incubation at room temperature, the mixture was added to cells maintained in 750 μL of complete DMEM. After 24 hrs incubation, HEK293 cells were used for transport assays. To verify over-expression in both HEK293-F and in HEK293 cells, proteins were extracted from HEK293-F and HEK293 cell pellets, solubilized with 1% C_12_E_8_, in the presence of protease inhibitor, quantified using the Lowry-Folin colorimetric method^19^ and subjected to WB analysis.

#### Over-expression and purification of recombinant hLAT1 and hxCT proteins

The proteins hLAT1WT and mutants F252A and K204Q were overexpressed in *E. coli* Rosetta(DE3)pLysS and purified on ÄKTA Start using affinity chromatography, as previously described.^23^ In brief, the supernatant from *E. coli* solubilization was loaded on a His Trap HP column (5 mL Ni Sepharose) pre-equilibrated with 10 mL of a buffer composed of 20 mM Tris HCl pH 8.0, 10% glycerol, 200 mM NaCl, and 0.1% sarkosyl. After sample loading, the column was washed with 10 mL of a washing buffer (buffer A) composed of 20 mM Tris HCl pH 8.0, 10% glycerol, 200 mM NaCl, 0.1% DDM, and 3 mM DTE. The WT or mutant proteins was eluted with 15 mL of washing buffer (buffer A) added with 400 mM imidazole (buffer B); fractions of 1 mL were eluted. Then, 2 mL of the purified hLAT1, were loaded onto a PD-10 desalting column to remove imidazole and NaCl. The desalting buffer was composed of 20 mM Tris HCl pH 8.0, 10% glycerol, 0.1% DDM, and 10 mM DTE; 2 mL of desalted protein was collected for downstream functional assay. The protein hxCT was overexpressed in Rosetta(DE3)pLysS and purified using Ni^2+^ affinity chromatography, as previously described.^64^ In brief, the *E. coli* pellet was solubilized by adding 8 M urea, 500 mM DTE, 10% sarkosyl, and 1 mM glutamate and mixed for 30 minutes in a fixed angle rotator for tubes. Then, renaturing buffer (A) containing: 0.1% sarkosyl, 200 mM NaCl, 10% glycerol, 1 mM glutamate, and 20 mM Tris HCl at pH 8.0 was added and the rotation continued for 30 minutes in the same conditions. After this time, the sample was centrifuged (12000g, 10 minutes, 4°C), and the supernatant was added to a His select Ni^2+^ affinity gel column (0.5 x 7.5 cm; 3 mL= 1 Column Volume) equilibrated with 10 column volumes of a renaturing buffer A, and then mixed for 1 hour at 4°C in a fixed angle rotator for tubes. Then, the protein resin mix was transferred into a column and packed by gravity. Then, 5 mL of washing buffer (0.1 % C_12_E_8_, 200 mM NaCl, 10% glycerol, 1 mM glutamate, 5 mM DTE, 20 mM Tris HCl at pH 8.0) were added to remove unbound proteins. A second washing step was performed with 10 mL of the same buffer added with 10 mM imidazole. Then, 5 mL of washing buffer (0.3% C_12_E_8_, 200 mM NaCl, 10% glycerol, 1 mM glutamate, 5 mM DTE, 20 mM Tris HCl at pH 8.0) prepared with 10 mM imidazole was added to increase detergent concentration required for protein solubility. The elution was performed using 8 mL of elution buffer composed of: 0.3% C_12_E_8_, 200 mM NaCl, 10% glycerol, 1 mM glutamate, 5 mM DTE, 20 mM Tris HCl at pH 8.0, and 500-mM imidazole. The purified xCT was passed through a PD-10 column for removing imidazole using a buffer composed of 0.3% C_12_E_8_, 200 mM NaCl, 10% glycerol, 5 mM DTE, 1 mM glutamate, 20 mM Tris HCl at pH 8.0.

#### Liposome preparation

For removing calcium phosphate from phospholipids, 3 mM EDTA was added to 10% egg yolk phospholipids and incubated for 15 min at room temperature. Then chloroform was added in a 1:1 ratio with phospholipids and the solution was centrifuged for 15 min at 12000 g, at 4°C using a fixed angle rotor. The supernatant containing clean phospholipids in chloroform is evaporated by rotavapor at 40°C. Then, 7.5% of cholesterol was added to phospholipids film obtained by rotavapor (except where different indicated) and then was dissolved with chloroform. After incubation under rotatory stirring (30°C 15 min 750 rpm) solution was dried using rotavapor. The lipid film was resuspended in water (10% final concentration) and single bilayer liposomes were prepared by two sonication cycles of 1 min (1 pulse ON and 1 pulse OFF, 40 W) with a Vibracell VCX-130 sonifier as previously suggested.^70^

#### Reconstitution of the hLAT1 and hxCT transporters into proteoliposomes

The desalted hLAT1 and hxCT were reconstituted by removing the detergent from mixed micelles containing detergent, protein, and sonicated phospholipids by incubation with Amberlite XAD-4 in a batch-wise procedure as previously described.^23^^,^^64^ In brief, the mixture for hLAT1 reconstitution was composed of 7 μg purified protein, 100 μL of 10% C_12_E_8_, 100 μL of sonicated liposomes prepared with 7.5% cholesterol, 10 mM histidine (except where differently specified in the figure legends), 10 mM DTE, 4 mM ATP (except where different indicated) and PBS without NaCl pH 7.0 in a final volume of 700 μL. Amberlite XAD-4 (0.5g) was added to this mixture and incubated for 90 min at 1200 rpm on a thermoshaker incubator at 23°C.^40^ The cell lysates derived from HEK293 FreeStyle overexpressing or not hLAT1 were reconstituted with the same procedure above-described with the sole difference that 150μg of total lysate was added to the reconstitution mixture. For the hxCT reconstitution, the mixture was composed of 7 μg purified protein, 100 μL of 10% C_12_E_8_, 100 μL of sonicated liposomes prepared with 7.5% cholesterol, 10 mM DTE and PBS without NaCl pH 7.0, in a final volume of 700 μL. Amberlite XAD-4 (0.5g) was added to this mixture and incubated for 90 min at 1200 rpm on a thermoshaker incubator at 23°C.^64^

#### Transport measurement in proteoliposomes

After the batch-wise procedure, 600 μL proteoliposomes were passed through a Sephadex G-75 column (0.7 cm diameter×15 cm height) equilibrated with a buffer containing PBS without NaCl pH 7.0 and 10 mM sucrose to balance osmolarity (unless where differently specified in the figure legends). Then, eluted proteoliposomes were divided into 100 μL aliquots for transport assay. Transport was started by adding 5 μM [^3^H]-histidine to proteoliposomes containing 10 mM histidine (unless where differently specified in the figure legends); according to the stop inhibitor method, the blank sample is prepared by adding 5 mM of 2-Amino-2-norbornanecarboxylic acid (BCH) (Napolitano et al.,2017). To remove the external (not taken up) radioactivity, 100 μL of each sample was passed through a Sephadex G-75 column (0.6 cm diameter×8 cm height). Samples were eluted with 1 mL 50mM NaCl in 4 mL of Pico-Fluor Plus and radioactivity was counted. To evaluate nmol/mg of substrate taken up by proteoliposomes, radioactivity counted for each sample was normalized for protein amount in mg (see other methods) and for the total radioactivity added to each sample, containing known [^3^H]-histidine nmoles. The β-counter (Tricarb 2810 TR) was daily calibrated using the internal radioactivity standards employing the software from Perkin Elmer. For copper determination in proteoliposomes, hLAT1 was reconstituted as described in the above paragraph and transport was started by adding 5 μM histidine to proteoliposomes in the presence or the absence of copper(II)chloride (as indicated in the figure legends). To remove the external (not taken up) histidine and copper, 100 μL of each sample were passed through a Sephadex G-75 column (0.6 cm diameter×8 cm height). Samples were eluted with 1 mL 50 mM NaCl and five samples were pulled together; C_12_E_8_ 0.25% was added to disrupt proteoliposomes for copper quantification. The samples were subjected to ICP analysis described below. In all the experiments, copper(II)sulfate, copper(II)chloride and histidine were solubilized and diluted in PBS without NaCl pH 7.0, preincubated 30 minutes before starting the transport assay.

#### Transport measurement in intact cells

HEK293 cells, transiently transfected as previously described were used for transport assay 24 hrs after transfection. Cell medium was replaced by HBSS transport buffer (5 mM KCl, 1 mM CaCl_2_, 0.4 mM MgSO_4_, 0.5 mM MgCl_2_, 0.3 mM Na_2_HPO_4_, 0.4 mM KH_2_PO_4_, 6 mM D-glucose, 4 mM NaHCO_3_) at 37°C. After washing, 450 μL warm HBSS was added for the transport assay in the presence of 40 μM [^3^H]-histidine and 20 μM copper(II)sulfate. The transport was stopped after 1 min by rinsing the cells three times with the same ice-cold transport buffer (500 μL per well per rinse). Then, cells were solubilized in 500 μL of 1% TX-100 solution, and 400 μL cell extracts were counted for radioactivity. The remaining 100 μL in each well was used for measuring total protein concentration. LAT1 specific transport was evaluated by subtracting the transport values of each condition from those deriving from blank, i.e., samples treated with the well-known LAT1 specific inhibitor BCH, used at 10 mM and added at time zero together with radiolabelled [^3^H]-histidine in the transport buffer.

#### Preparation and characterization of the Cu(His)_2_ complex

At pH of 7.0-7.5, a neutral, monomeric Cu(His)_2_ species featuring the histidinate monoanion readily forms when reacting L-histidine with a variety of copper(II) salt, such as, for instance, CuSO_4_, CuCl_2_, Cu(NO_3_)_2_, in a 2:1 stoichiometric ratio, regardless of the specific copper source, as confirmed by absorption spectroscopy. To crystallize the complex, we used CuSO_4_ as the copper source and followed the literature crystallization procedure.^43^ FT-IR spectra (4000 – 500 cm^-1^) were recorded on a Perkin-Elmer Spectrum One FT-IR spectrometer as KBr pellets. UV/vis absorption spectra were obtained with a PerkinElmer Lambda 900 spectrophotometer, using quartz cuvettes of a 1 cm path length. Powder X-ray diffraction patterns were acquired with a Bruker D2-Phaser equipped with a Cu Kα radiation (λ = 1.5418 Å) and a Lynxeye detector, at 30 kV and 10 mA, with a step size of 0.01°(2θ) between 5 and 40°(2θ). Single-crystal unit cell checks were performed at room temperature with a Bruker-Nonius X8-APEXII CCD area detector system by using graphite monochromated Mo-Kα radiation (λ = 0.71073 Å).

#### Docking analysis

LAT1 inward open conformation with cholesterol and ATP^23^ was used for docking analysis. AutodockVina v1.1.2 (The Scripps Research, La Jolla, California, CA, USA) was used to analyse copper-histidine-binding site in the LAT1 transporter using the docking procedure. To prepare the structure for the subsequent steps, polar hydrogens and Kollman charge were added. Ligands were prepared by adding hydrogens and charge. After ligand and receptor preparation, a grid box was generated on the whole internal cavity of the protein. The box size was 19.96 × 32.45× 23.18 Å (x, y, and z), with a spacing of 0.5 and an exhaustiveness value of 8. Thirty poses from 3 docking simulations for each dataset (i.e. the Cu(his)_2_ complex with the pendant imidazole ring in the starting *a* or *b* orientation, see Figure 5D) were carried out. A more negative docking score pose was chosen. Molecular graphics and visualization of docking results were performed with the UCSF Chimera v.1.14 software (Resource for Biocomputing, Visualization, and Informatics, University of California, San Francisco, CA, USA).

#### ICP-mass

The copper contents of proteoliposomes prepared as described in the previous paragraph were determined by a quadrupole Inductively Coupled Plasma-MassSpectrometer (ICP-MS, PerkinElmer, model ElanDRCe). In each analytical sequence, procedural blanks and reference materials were included. The limit of detection was set as three times the standard deviation of the procedural blanks, and its value is 0.03. The precision and accuracy of the applied analytical method were estimated on water Certified Reference Material (NIST1643f); the certified Cu concentration is 21.66 ± 0.71 μg∗L^−1^, and the data (mean±SD) obtained was 22.15 ± 0.22 μg∗L ^−1^.

#### Data analysis

All experimental data are derived from the mean of three independent experiments and results are expressed as means ± SD. Kinetic parameters were derived from data fitting in Michaelis-Menten equation using Grafit v 5.0.13 software (Erithacus Software, West Sussex, UK). Comparisons between the two groups were performed with the two-tailed Student’s unpaired t-test for p< 0.05 and p<0.01. For multiple comparisons, non-parametric Kruskal-Wallis test was employed.

#### Other methods

The amount of purified recombinant hLAT1 WT was estimated from stain-free 12% SDS–PAGE gels by using the Chemidoc imaging system equipped with Image lab software (Bio-Rad) as previously described, in absolute quantification using BSA as a standard.^40^ Immunoblotting analysis was performed using monoclonal 1:1000 anti-HA (Merck), 1:1000 anti-FLAG (Merck) and 1:5000 anti-actin (Merck), incubated overnight in 3% BSA under shaking at 4°C. The 1:10000 anti-mouse secondary antibody was incubated for 1h in 1% BSA under shaking at room temperature. The immunoblot was revealed by chemiluminescence assay (SuperSignal™ West Femto Maximum Sensitivity Substrate) using the Chemidoc imaging system equipped with Image Lan software (Bio-Rad).

## Data Availability

• All data reported in this paper will be shared by the lead contact upon request. • This paper does not report original code. • Any additional information required to reanalyze the data reported in this paper is available from the lead contact upon request.
