# Supplementary material for: LAT1 (SLC7A5) catalyzes copper(histidinate) transport switching from antiport to uniport mechanism
Source: iScience. 2023 Aug 26;26(10):107738. doi: 10.1016/j.isci.2023.107738 (PMC10492218; doi:10.1016/j.isci.2023.107738)
Supplement: Document S1. Figure S1 [file mmc1.pdf]

iScience, Volume *26*

Supplemental information

LAT1 (SLC7A5) catalyzes copper(histidinate) transport switching from antiport to uniport mechanism

Raffaella Scanga, Mariafrancesca Scalise, Nadia Marino, Francesco Parisi, Donatella Barca, Michele Galluccio, Chiara Brunocilla, Lara Console, and Cesare Indiveri

Supplementary information

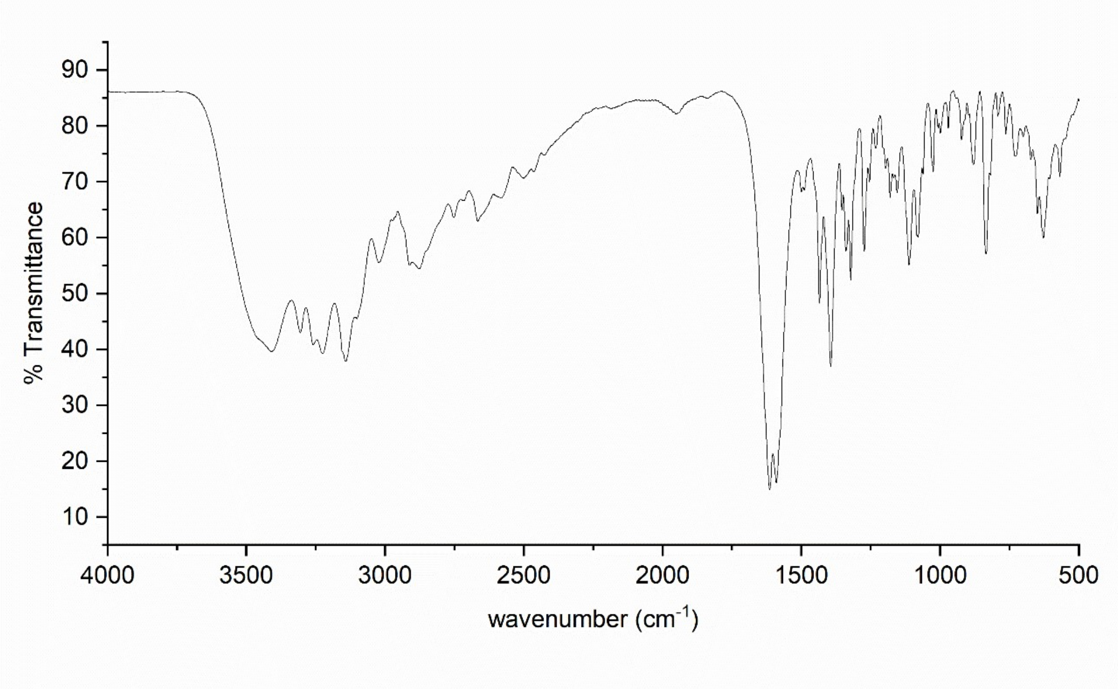

Supplementary Figure 1 related to Figure 5. FI-TR spectrum.

FT-IR spectrum (KBr pellets, transmittance mode, 4000-500 cm-1) of the complex [Cu(His)$_2$]·1.5H_2O obtained in this work.
